# Green Synergy: Interplay of corporate social responsibility, green intellectual capital, and green ambidextrous innovation for sustainable performance in the industry 4.0 era

**DOI:** 10.1371/journal.pone.0306349

**Published:** 2024-08-08

**Authors:** Zohaib Zahid, Jijian Zhang, Muhammad Asim Shahzad, Muhammad Junaid, Archana Shrivastava

**Affiliations:** 1 School of Finance and Economics, Jiangsu University, Zhenjiang, China; 2 School of Management, Jiangsu University, Zhenjiang, China; 3 College of Business Management, Institute of Business Management, Karachi, Pakistan; 4 Business Communication, Birla Institute of Management Technology, Greater Noida, U.P, India; Hubei Engineering University, CHINA

## Abstract

This study delves into the interconnections among corporate social responsibility, green intellectual capital, green ambidextrous innovation, and sustainable performance, particularly in the context of Industry 4.0 and sustainability. A questionnaire-based survey was conducted, and a sample of 317 small and medium enterprises was collected. Using Partial Least Squares Structural Equation Modeling in Smart-PLS v4, the findings reveal a significant relationship between corporate social responsibility and sustainable performance, with green intellectual capital and green ambidextrous innovation serving as mediating factors. Moreover, the study highlights the moderating role of Industry 4.0 among green intellectual capital and green ambidextrous innovation with sustainable performance. These findings may guide the managers in designing and implementing CSR strategies beyond compliance and contributing to competitive advantage through green intellectual capital and green ambidextrous innovation for business success in the era of Industry 4.0.

## 1. Introduction

In today’s rapidly evolving business world, prioritizing sustainable development has become crucial for organizations [1]. Companies are actively seeking innovative strategies to align their operations with sustainability goals owing to environmental concerns and the demand for responsible business practices [2]. Extensive research is being conducted on various aspects of green practices, such as green innovation (GI) [3–5], green human resource management [6, 7], green supply chain management [8], green purchasing strategies [9], and transition to circular economy [10]. Companies have recognized the importance of corporate social responsibility (CSR) as a strategic approach that extends beyond purely economic considerations [11]. Engaging in corporate sustainability activities and complying with ecological regulations generates a harmonious situation, leading to enhanced sustainable performance [12]. The growing prominence on CSR and environmental responsibility offers an opportunity for companies to enhance their intellectual capital [13]. Intellectual capital has been extensively studied and recognized as critical in driving sustainable performance (SP). However, its green aspect, known as green intellectual capital (GIC), remains relatively unexplored [3, 14, 15]; investing in GIC fulfills environmental management standards and provides a competitive advantage [16]. It provides a distinct perspective on how organizations can utilize their intangible resources to generate value while addressing environmental concerns and promoting sustainable practices [17]. By harnessing GIC, companies can develop competitive advantages to improve their environmental performance and contribute to sustainable development [18]. GIC represents a collection of impalpable assets, knowledge, and competence encompassing ecological preservation and green innovation [19].

Green innovation involves developing and adopting new or improved products, operations, technologies, strategies, and managerial approaches contributing to sustainability [20, 21]. It emphasizes integrating environmentally friendly practices throughout the value chain to reduce resource consumption, minimize pollution, and enhance overall environmental performance [22]. Organizations must develop and implement GI strategies prioritizing energy efficiency, contamination avoidance, and environmental condition improvement [17]. This requires a balanced focus on both exploitative and exploratory innovations, a combo of green ambidextrous innovation (GAI) (GAI is a strategy that incorporates environmental sustainability with the dual capability of exploiting existing competencies and exploring new opportunities.) [3]. Green exploitative innovation focuses on refining and applying existing knowledge and technology, while green exploratory innovation focuses on new knowledge opportunities and technologies [23]. Although previous studies have examined the relationships between CSR, innovation, and SP [3, 15, 24, 25], there remains a significant research gap in understanding how integrating GIC and GAI facilitated by the Industry 4.0 ecosystem (I4.0ES) can enhance SP. The existing literature recognizes CSR’s importance as a strategic practice for building relationships with stakeholders and acquiring external knowledge [26]. However, limited consciousness has been paid to the mediating role of GIC and GAI in linking CSR with SP.

Further, CSR is a significant predictor of small and medium enterprises (SMEs) performance [27, 28]. Literature has confirmed that CSR plays a vital role in achieving SP [29]. The researchers paid scant attention to CSR to measure SP. This study aims to evaluate SP through GIC and GAI. Industrial practitioners and scholars have concentrated on environmental strategy [30]. Kraus [31] found that environmental strategy (e.g., innovative preventive practices and eco-efficient practices) is positively associated with SP. Walker [32] asserted that a proactive environmental strategy is considered a significant factor in examining SME firms’ performance. The motivation behind this study is that researchers have paid less attention to CSR to determine SP in SME firms in China with the mediating role of GIC and GAI. Moreover, I4.0ES is characterized by the integration of smart technologies, including the Internet of Things (IoT), Big Data, Cloud Computing, and Cyber-Physical Systems (CPS), which opens fresh opportunities for environmentally friendly innovation [33, 34]. The I4.0ES establishes a digital environment that enables the autonomous regulation of machines. It enables seamless integration and facilitates the diffusion of technologies across the value chain [35, 36].

Prior literature extensively discusses issues related to GAI and sustainable performance [31, 32], but none have explored CSR, GAI and I4.0ES. Building upon previous literature, our study incorporates corporate social responsibility, green intellectual capital, green ambidextrous innovation, sustainable performance and Industry 4.0—forming an integrated framework to examine their relationships in one conceptual framework for SMEs. It is necessary to identify the factors that may interact with an SME firm’s CSR and GIC with innovation and performance. As mentioned above, earlier studies explored the significant relationship between CSR and SP, but the intervening variables were not well-explored, necessitating the use of GIC, GAI, and I4.0ES as mediating and moderating variables, respectively. Despite this, the existing literature has failed to determine the role of I4.0ES among GIC, GAI, and SP. Therefore, this study aimed to fill this gap by addressing how CSR affects SP and how GIC and GAI mediate between the studied variables. The study also explores how the moderating effect of the I4.0ES can enhance SP. This study answers the following questions:

1. What is the impact of CSR on sustainable performance?
2. What is the role of green intellectual capital and green ambidextrous innovation in driving sustainable performance?
3. Does the I4.0ES enhance the relationship between green intellectual capital and green ambidextrous innovation?
4. How do green intellectual capital and green ambidextrous innovation mediate the relationship between CSR and sustainable performance?

The main contribution of this study is to examine the interdependence between CSR, GIC, GAI, and sustainable performance in the context of I4.0ES. By integrating these key aspects and analyzing their fused effects, this study provides novel insight into how organizations can use CSR practices to build intellectual capital, promote innovation, and achieve sustainable performance. This study also provides insightful recommendations for managers looking to develop and implement CSR strategies that not only comply with regulatory requirements, but ultimately enhance competitiveness and promote sustainable performance in the current era of I4.0ES. The details of theoretical and practical contributions are mentioned in Section 6.

The rest of the paper has the following sections. The theoretical framework and development of hypotheses are established in Section 2. The research methodology, sample selection process, and data analysis strategies are covered in Section 3. The data analysis and results are presented in Section 4. The discussion and conclusion are discussed in Section 5. Theoretical and practical significance are presented in Section 6. Limitations and future research avenues are reported in Section 7.

## 2. Theoretical framework and hypotheses development

### 2.1 Theoretical underpinning

This study establishes its theoretical foundation on the NRBV and stakeholders’ theory to support the study’s conceptual framework and hypotheses. According to RBV theory, organizational resources and capabilities are significant in attaining a competitive advantage [37]. Moreover, NRBV theory is the extended form of RBV theory, which hypothesizes that firms can gain sustained competitive advantage in responding to answer issues about the natural environment [38]. NRBV posits that firms’ unique natural resources can provide a sustainable competitive advantage [37, 38]. Researchers used NRBV theory to measure SME firms’ performance by focusing on using CSR environmental, social, and economic aspects [39–42]. In contrast, prior research also used contingency theory for environmental strategy and environmental managerial performance [43], stakeholder theory for CSR and economic performance [44], and environmental performance [45]. However, there has been relatively less emphasis on measuring SP through CSR, GIC, GAI, and I4.0ES by integrating NRBV and stakeholders’ theory.

Therefore, this study used CSR (economic dimension, social dimension, and environmental dimension), GIC, and GAI to enhance sustainable performance considering the NRBV theory. CSR is essential to a firm’s resource portfolio [46]. Firms can enhance their competitive advantage and SP by incorporating CSR practices [1]. CSR activities improve a firm’s reputation and stakeholder relationships, which are valuable intangible assets in the NRBV framework [47]. Companies can leverage CSR as a resource capability to strengthen stakeholder relationships, create long-term value, and gain a competitive advantage [48]. Conversely, GIC mentions the knowledge, expertise, and intellectual property related to environmentally friendly practices and technologies within organizations [3, 49]. Firms can utilize GIC to harness their specific environmental knowledge and capabilities, fostering innovative and sustainable solutions [20]. Similarly, GAI serves as a manifestation of NRBV within the domain of sustainability. GAI refers to firms’ ability to explore and exploit green opportunities simultaneously, facilitating the integration of environmental concerns into innovation processes [19]. By adopting GAI, firms can balance exploring new environmentally friendly technologies and practices with exploiting existing green capabilities, leading to enhanced SP [50, 51].

**Fig 1:**
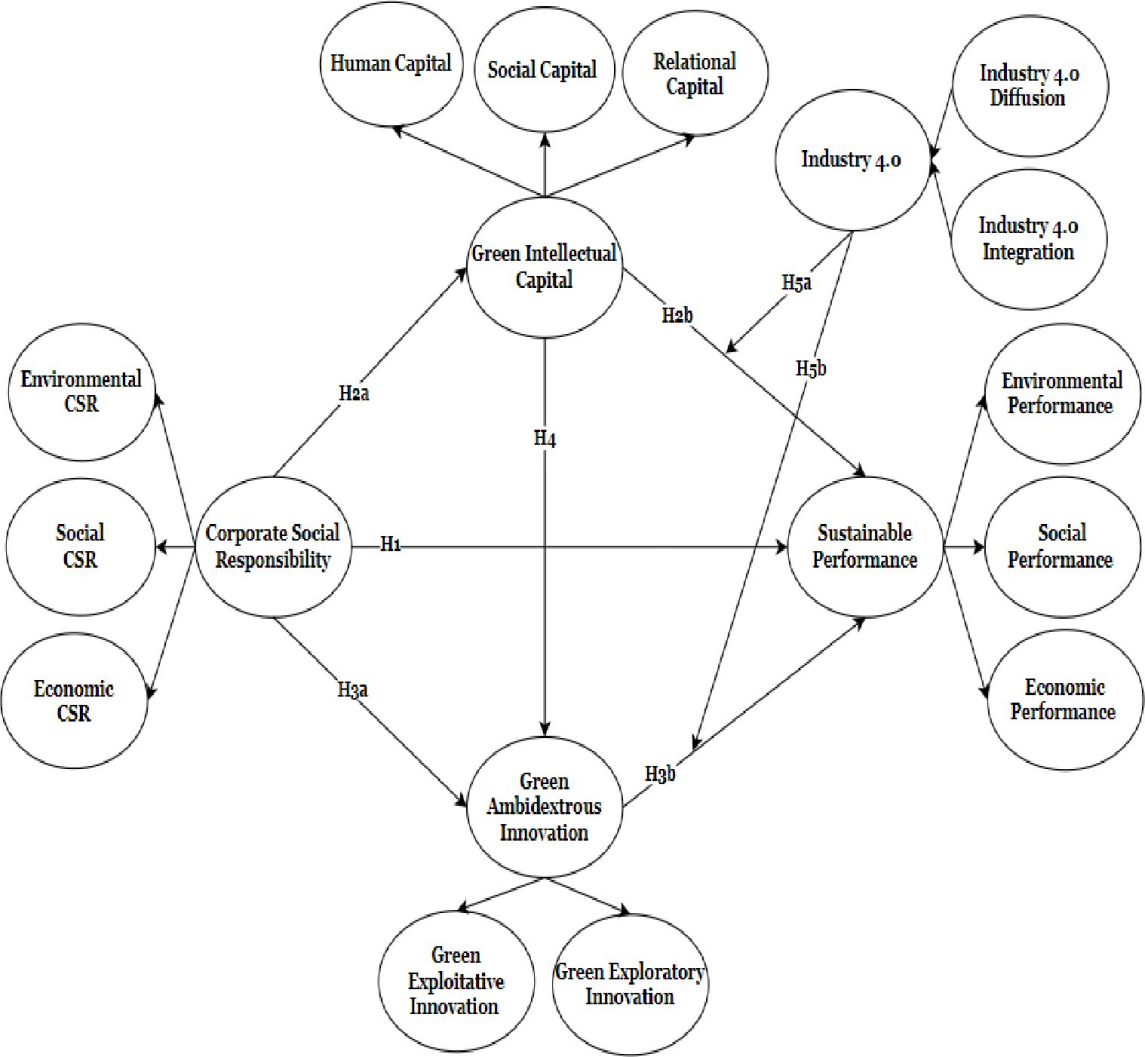
Conceptual framework.

Furthermore, the stakeholders’ theory provides a contextual lens to understand the external pressures and norms that shape firms’ behavior and decision-making processes [52]. The I4.0ES, as a moderating variable in this study, represents the institutional environment within which firms operate. The I4.0ES encompasses interconnected technologies, processes, and stakeholders that enable digital transformation and advanced manufacturing practices [35, 36]. Considering the institutional context, integrating these theories in this study helps investigate how the I4.0ES may influence the relationships between GIC, GAI, and SP. We have developed a conceptual framework based on the overhead arguments, as shown in Fig 1.

### 2.2 Corporate social responsibility and sustainable performance

CSR has gained significant attention as organizations face growing pressure to address environmental, social, and economic challenges [53]. Buyers’ increasing urge for environmentally conscious products and services has prompted the emergence of eco-entrepreneurship, which aims to proactively mitigate environmental impacts and introduce eco-friendly innovations across industries [54, 55]. Stakeholders, including customers, employees, competitors, and governmental entities, expect organizations to take a strong stance on social and environmental issues, further emphasizing the importance of CSR [56].

CSR encompasses the strategic crafting of policies, decision-making processes, and actions that generate value for society [57]. It encompasses three fundamental dimensions: economic, social, and environmental [58]. Applied research highlights the positive impact of CSR on performance; much of the research has focused on economic performance [59]. Nonetheless, there is a need for comprehensive measurement and evaluation of CSR’s effects on all three dimensions of SP [26]. Organizations nowadays recognize the importance of incorporating social and environmental indicators into their performance measurement. Firms can effectively depict and measure the true value of CSR initiatives by integrating these indicators [60]. This holistic approach provides a broader and more stable view of corporate wealth and highlights the organization’s proficiencies in creating future environmental value [61]. Embedding a "social and environmental" perspective into performance measurement aligns with the principles of corporate sustainability, where environmental and social concerns are prioritized [62]. Companies with a robust measurement system for SP are likely to reap the non-monetary benefits from CSR practices [63].

Therefore, those organizations that actively engage in CSR activities experience enhanced SP [64]. Obsessing social and environmental indicators alongside other performance dimensions can maximize the benefits derived from CSR practices [65]. This study proposes the following hypothesis based on previous literature and the significance of CSR in today’s business realm.

***H1***: *CSR has a positive relationship with sustainable performance*.

### 2.3 Corporate social responsibility and green intellectual capital

In today’s business landscape, CSR is increasingly important in promoting responsible business practices [1]. CSR entails an organization’s proactive participation in endeavors that advance society and the environment [66]. As environmental concerns gain prominence, organizations are increasingly expected to address these issues and integrate them into their core business strategies [67, 68]. One area of interest in the field of CSR is its relationship with GIC, which refers to the intangible assets linked to green innovation [13]. GIC aids firms in harnessing their intellectual resources to achieve sustainable development goals (SDGs) [17].

According to Chang and Chen [69], CSR initiatives positively influence GIC. CSR practices such as environmental consciousness and responsible resource management foster the accumulation of green knowledge. GIC is essential to provide one with a competitive advantage and aids in encouraging long-term performance. Those firms that actively adopt CSR practices and cultivate GIC together gain a competitive edge and SP together [70]. Based on these justifications, this study suggests the following hypothesis:

***H2a*:** *CSR positively influences green intellectual capital*.

### 2.4 Green intellectual capital and sustainable performance

GIC is crucial in fostering sustainable business practices in different parts of the organization. According to Yong [71], GIC is key to cultivating green human resource management. Strong emphasis on GIC promotes HR strategies that integrate environment-friendly approaches. Integration between GIC and HR practices contributes to overall sustainability. According to Erinos and Yurniwati [72], GIC positively affects firms’ performance. This means that firms that encourage employees to cooperate and network are more likely to achieve SP. Therefore, GIC encourages collaboration and knowledge sharing, which seizes sustainable opportunities. Prior studies emphasize the favorable links between GIC and SP [3, 13, 17].

GIC provides businesses the tools to comply with strict environmental standards, adapt to changing consumer demands for environmentally friendly goods and services, and create value through sustainable practices [19]. The positive effects observed in previous studies regarding green structural capital, competitive advantage, environmental competence, commitment-related activities, and green product innovation further underscore the role of GIC in driving SP [25, 73, 74]. Firms investing in strong green structural capital are more likely to achieve higher SP [17].

Considering these findings, we posit a positive connection between GIC and SP. Organizations that cultivate and leverage intellectual capital are more likely to achieve sustainable outcomes. Based on the above arguments, this study develops the following hypotheses.

***H2b*:** *Green intellectual capital has a positive relationship with sustainable performance*.
***H2c*:** *Green intellectual capital mediates the relationship between CSR and sustainable performance*.

### 2.5 Corporate social responsibility and green ambidextrous innovation

CSR is essential for businesses to access outside information as a strategic approach for establishing and managing connections with various stakeholders [75]. GI and GAI are two examples of how CSR and GI are related in literature. Green innovation creates novel or enhanced goods, methods, or technology advantageous to ecological and financial concerns [76, 77]. Compared to conventional innovation, it is distinguished by reliance on a variety of knowledge and integration of technology elements, which frequently brings unpredictability and disruption [77, 78]. This situation highlights the value of open innovation and demonstrates how GI thrives on R&D collaboration with stakeholders and outside knowledge sources [79]. Existing research indicates that collaboration with stakeholders and engagement with external networks might help organizations acquire new knowledge and information more efficiently, ultimately improving their capacity for innovation [80].

According to the ambidextrous innovation idea, green innovation has two forms: exploratory and exploitative [3]. Exploratory green innovation involves firms’ environmental efforts to identify or generate new knowledge, expanding the existing knowledge base through extensive stakeholder linkages [81]. In contrast, exploitative green innovation concerns businesses’ efforts to expand or enhance the current body of knowledge through close associations with certain stakeholders [19]. Drawing on NRBV, CSR is assumed to influence GAI. Companies implementing more CSR conformity tend to benefit from constructing external networks with diverse stakeholders. This enables the acquisition of cutting-edge and diversified knowledge, broadening firms’ human and relational capital and ultimately promoting GAI [82]. Additionally, associating with diverse stakeholders through CSR allows firms to gather competitive market knowledge, reducing the risk associated with GAI [83]. Based on the above discussion, this study proposes the following hypothesis.

***H3a*:** *CSR has a positive relationship with green ambidextrous innovation*.

### 2.6 Green ambidextrous innovation and sustainable performance

GAI refers to organizations’ simultaneous pursuit of exploitative and exploratory green innovation practices [84, 85]. Exploitative innovation involves refining and optimizing existing green technologies, products, and processes, while exploratory innovation focuses on developing novel and disruptive green solutions [81]. By engaging in GAI, companies can effectively address environmental challenges while leveraging existing capabilities and exploring new opportunities for sustainable development [82]. GAI is instrumental in driving SP for several reasons. On the one hand, organizations use exploitative innovation to optimize their current green technologies, products, and processes, leading to improved resource efficiency, reduced environmental impact, and enhanced operational performance [86]. This allows companies to address existing sustainability challenges and meet regulatory requirements effectively.

In contrast, through exploratory innovation, companies try to advance green products, services, and technologies that align with emerging environmental trends and customer demands [87]. Exploratory innovation enables organizations to identify and capitalize on new market opportunities, foster eco-friendly consumer behavior, and create a positive environmental impact [3]. Companies can stay ahead of the competition by continuously exploring new knowledge, pushing green innovation boundaries, and achieving sustainable growth. Integrating exploitative and exploratory practices encourages knowledge sharing, cross-functional collaboration, and the development of a learning-oriented mindset [82]. This makes it easier for the organization to acquire and spread green knowledge, enabling employees to respond to environmental changes and drive SP effectively [3]. Based on the above justifications, this study develops the following hypotheses.

***H3b*:** *Green ambidextrous innovation has a positive relationship with sustainable performance*.
***H3c*:** *Green ambidextrous innovation mediates the relationship between CSR and sustainable performance*.

### 2.7 Green intellectual capital and green ambidextrous innovation

GIC encompasses the knowledge, skills, and intangible assets related to sustainability within an organization [88]. GIC is vital for GAI, as it involves exploitative and exploratory strategies. Through exploitative and exploratory innovation, firms develop existing capabilities and explore new opportunities [17]. GIC provides organizations with the necessary intellectual resources to develop and implement green innovation practices [3]. GAI helps firms refine and apply existing environmental knowledge and practices (exploitative innovation) and explore new avenues for green product development and market expansion (exploratory innovation) [19].

Furthermore, the literature throws light on the role of the arrangement of resources in stimulating GAI. Wang and Juo [17] state that effective resource orchestration, including the management and integration of GIC, can facilitate exploitative and exploratory green innovation activities. By harmonizing different forms of GIC, such as green human, structural, and relational capital, organizations can achieve a synergistic effect that enhances their ability to promote existing capacities and explore new opportunities [26]. Green human capital refers to the knowledge, skills, and expertise of individuals in an organization contributing to environmental sustainability. It involves employees’ awareness, competencies, and commitment to green practices. Green structural capital encompasses the organizational structures, processes, and systems that support green innovation initiatives. This includes establishing a dedicated R&D efforts blueprint promoting sustainability. Green relational capital refers to the relationships, collaborations, and networks the organization forms with external stakeholders, such as customers, suppliers, and government agencies, to foster green innovation and sustainability [17]. Organizations with higher levels of GIC are more likely to engage in GAI activities, leading to improved SP. This study creates the following hypothesis based on the justifications mentioned above.

***H4*:** *Green intellectual capital has a positive relationship with green ambidextrous innovation*.

### 2.8 Moderating role of industry 4.0 ecosystem

As recent literature reviews highlight [79–81], several research streams can be identified in the I4.0ES domain. These streams can be distinguished according to the scope of applications of digital technologies [89], which can be either internal or external information that affects SP. The I4.0ES refers to integrating and implementing technology-enhanced production processes [36]. It is characterized by integrating smart technologies such as the IoT, BD, CC, CPS, etc. [90]. The I4.0ES consists of two dimensions: technology integration and diffusion [91]. Technology integration encompasses horizontal, vertical, and end-to-end integration. Horizontal integration focuses on the connections in the value chain, streamlining the flow of data and products between the organization and its customers [92, 93]. Vertical integration enables adaptable manufacturing systems and networks to support smart functioning and responsiveness to client orders and market demands. End-to-end integration involves the design and construction of products, incorporating consumer demands and enabling customization [94]. However, many contributions to I4.0ES mainly adopt a firm-level perspective [33, 95–98], with literature highlighting that I4.0ES is a wider phenomenon that goes beyond the firm’s boundaries and requires the contribution of other factors, such as GIC, which must adapt their work and mission [99, 100]. A useful lens to explore I4.0ES from such a perspective is the innovation ecosystem concept to enhance a firm’s sustainability.

The five characteristics of innovation precedence, harmony, intricacy, detectability, and reliability—impact the diffusion of I4.0ES across the firm and its ecosystem [101]. Relative advantage refers to the preference for new technology over existing ones, while compatibility relates to the alignment with current processes and principles [102]. Complexity reflects new technology’s learning and usability challenges, and trialability refers to the ease of experimentation before extensive use [34]. Rong [103] was among the early contributors to adopt the ecosystem concept for analyzing I4.0ES and its associated technologies. They identified six structural elements necessary to describe the evolution of an IoT-based business ecosystem, indicating that this latter is a complex network supported by different stakeholders. Similarly, Kahle [104] proposes a conceptual framework depicting the features that an innovation ecosystem must have to develop and offer smart products properly and identify the complementary capabilities needed in this context.

Overall, SMEs are more likely to leverage digital technologies to establish GIC and GAI to achieve higher economic and environmental performance in a dynamic environment. The I4.0ES plays a crucial moderating role in the relationships between GIC, SP, and GAI and SP. The I4.0ES can enhance the impact of GIC and GAI on SP by providing an enabling environment for implementing and utilizing GIC and GAI within the context of I4.0ES. Based on the above arguments, this study develops the following hypotheses.

***H5a*:** *Industry 4*.*0 ecosystem moderates the relationship between green intellectual capital and sustainable performance*.
***H5b*:** *Industry 4*.*0 ecosystem moderates the relationship between green ambidextrous innovation and sustainable performance*.

## 3. Methodology

This study employs an exploratory-quantitative approach to examine the relationships between CSR, GIC, GAI, I4.0ES, and SP. It seeks to enhance our understanding of the interplay among these factors and their potential contributions to sustainable development.

This study was conducted in accordance with the research ethics outlined in the APA’s Ethical Principles of Psychologists and Code of Conduct [105]. This study did not require an ethical review statement from the institutional review committee. An ethical statement is only required for studies employing specific research designs. Research permission was given by the organization. The data were provided to the researcher in an anonymized format and managed to ensure that no respondents could be identified. The study participants provided informed consent, confirming their understanding of the study’s purposes and voluntary participation. Participants were assured of the confidentiality of their identities and responses, which would be maintained to uphold academic integrity. This study outlines the research methodology to achieve our objectives in the following subsections.

### 3.1 Constructs, dimensions and sources

Table 1 presents the constructs, respective dimensions, and sources.

**Table 1:** Constructs and dimensions.

Constructs	Dimensions	Sources
Corporate Social Responsibility (CSR)	Environmental CSR Social CSR Economic CSR	Kraus [31]
Green Intellectual Capital (GIC)	Human Capital Relational Capital Social Capital	Chang and Chen [69]
Green Ambidextrous Innovation (GAI)	Green Exploitative Innovation Green Exploratory Innovation	Wang and Juo [17]
Sustainable Performance (SP)	Environmental Performance Social Performance Economic Performance	Yong [71] and Haseeb [106]
Industry 4.0 Ecosystem (I4.0ES)	Industry 4.0 Diffusion Industry 4.0 Integration	Pérez-Lara [93] and Arnold [107]

### 3.2 Population and sampling

This study collected data from SMEs located in China. The philosophical underpinning of this study was the deductive method, which is mainly appropriate to the positivist paradigm and allows the development of hypotheses and analytical testing at an acceptable probability level of expected results [108]. The study employed stratified random sampling techniques to ensure the representation of firms. This method ensures that the selection is unbiased and reflects a fair and representative sample of SMEs within those industries [109]. The G*Power software was utilized to determine the minimum required sample size [110]. Based on the G*Power test, which considers factors such as effect size, alpha level, power, and the number of predictors, a minimum of 295 participants was necessary to assess the variables’ impact.

### 3.3 Data collection process

This study incorporates an online questionnaire survey to examine the target population and collect data on SMEs. The questionnaire was carefully designed to ensure that it covered all target variables and that the measures it contained were valid and reliable. To improve the overall quality, reliability, and cross-cultural validity of the study questionnaire, we added a face validity test and conducted a comprehensive back-to-back translation (English Chinese-English) based on expert feedback. This study ensured that the instrument captured the necessary structure and provided useful data for analysis that relied heavily on these stages.

Initially, invitations were sent to approximately 580 randomly selected SMEs. Multiple efforts were made to get maximum response rates, including friendly reminders through referrals and professional networks. Consequently, multiple endeavors were undertaken via electronic mail to ensure participation in the survey. The study ensured that the participants possessed sufficient knowledge relevant to their job roles. After conducting two to three rounds of reminders, a total of 332 companies granted their consent to participate in this study. The data collection phase lasted approximately three months (March 2023—May 2023). Only one respondent from each SME was chosen to ensure data integrity and avoid multiple responses from the same SME. Throughout the data collection process, the anonymity of the respondents was strictly maintained to encourage open and honest responses. To maximize the response rate, unresponsive managers were contacted via email to encourage their participation in completing the survey. Among the returned questionnaires, a total of 317 respondents were evaluated as valid, culminating in a response rate of 95.48%. This percentage is deemed acceptable for research studies that employ survey methodology. The profile of the respondents and demographic information are presented in Table 2.

**Table 2:** Demographic information.

Particular	Description	Value	Percentage
Industry Sector	Textile	72	22.7
	Food and Beverages	96	330.3
	Beauty and Cosmetics	74	23.4
	Agriculture and Pesticides	46	14.2
	Leather and Tanneries	29	9.10
Gender	Male	213	67.0
	Female	104	32.9
Age of firms	≤5 years	80	25.3
	6–10 years	73	23.1
	11–20 years	78	24.6
	≥21 years	86	26.8
	Diploma Holder	52	16.4
Qualification	Undergraduate	79	25.0
	Graduate	117	36.7
	Postgraduate/Doctoral	58	18.3
	Others	11	3.48

## 4. Data analysis and results

This study chose the Partial Least Square Structural Modeling (PLS-SEM) using Smart-PLS v4 to validate the research model. PLS-SEM is appropriate for estimating large and complex models containing dozens of constructs with reflective-formative measures [111]. The model of this study is large and complex, including multidimensional constructs and reflective-formative measurement items, making PLS-SEM a suitable option. In addition, PLS-SEM facilitates theory development and emphasizes the model’s predictive power [112]. Moreover, PLS-SEM enables researchers to create more comprehensive and parsimonious models [113].

### 4.1 Common method bias

This study performed preliminary checks before evaluating the hypothesized model. Accordingly, the nonresponse bias and the Common Method Bias (CMB) were first examined. The study obtained data from a single source, so CMV was examined using Harman’s single-factor test [114–116]. The results show that extracting a first component explains 28% of the total variance, which is lower than the cut-off value of 40% [117–120]. Secondly, nonresponse bias was assessed by employing the independent sample t-test. Responses of early respondents were compared to late respondents to examine the presence of nonresponse bias [121, 122]. The insignificant p-values obtained from Leven’s test and t-test indicate the homogeneous characteristics of data collected from the respondents. Thus, no presence of nonresponse bias was found.

### 4.2 Assessment of the measurement model

This study incorporates a reflective-formative hierarchical framework commonly employed in previous empirical studies to establish higher-order models. The framework integrated variables constructed based on their dimensions, considered lower-order constructs (LOCs). Therefore, the LOCs were measured as reflective constructs [123]. On the other hand, each latent variable represented a higher-order construct (HOC) as its constituent constructs formed it. As a result, the HOCs were measured as formative constructs. The disjoint two-step approach was employed to validate the measurement model [113]. During the first stage, the internal consistency of the LOCs, including measures such as Cronbach’s Alpha, composite reliability (CR), average variance extracted (AVE), and discriminant validity, were assessed [113, 123, 124]. In the second stage, latent variable scores were obtained from the results generated by PLS analysis and added to the original data file to create the higher-order PLS path model and run the bootstrapping model. Table 3 represents the factor loadings (FL) values, CB-alpha, CR, and AVE. All the values exceed the threshold values.

**Table 3:** Convergent reliability and validity.

Constructs	Sub-Constructs	Items	Factor Loadings	Cronbach’s Alpha	CR	AVE
**CSR**	ECCSR	ECCSR1	0.808	0.898	0.924	0.710
		ECCSR2	0.799			
		ECCSR3	0.868			
		ECCSR4	0.905			
		ECCSR5	0.830			
	ENCSR	ENCSR1	0.758	0.738	0.836	0.560
		ENCSR2	0.739			
		ENCSR3	0.758			
		ENCSR4	0.739			
	SCSR	SCSR1	0.834	0.882	0.918	0.738
		SCSR2	0.879			
		SCSR3	0.870			
		SCSR4	0.852			
**GIC**	HC	HC1	0.729	0.800	0.870	0.626
		HC2	0.790			
		HC3	0.824			
		HC4	0.819			
	RC	RC1	0.743	0.824	0.883	0.655
		RC2	0.865			
		RC3	0.822			
		RC4	0.802			
	SC	SC1	0.834	0.757	0.847	0.584
		SC2	0.802			
		SC3	0.783			
		SC4	0.619			
**GAI**	GAI	GAI1	0.806	0.822	0.875	0.584
		GAI2	0.782			
		GAI3	0.749			
		GAI4	0.749			
		GAI5	0.732			
**SP**	ECP	ECP1	0.862	0.818	0.892	0.733
		ECP2	0.850			
		ECP3	0.857			
	ENP	ENP1	0.891	0.805	0.884	0.718
		ENP2	0.877			
		ENP3	0.768			
	SCP	SCP1	0.874	0.847	0.907	0.766
		SCP2	0.874			
		SCP3	0.878			
**I4.0ES**	I4.0 D	I4.0 D1	0.839	0.854	0.894	0.631
		I4.0 D2	0.622			
		I4.0 D3	0.814			
		I4.0 D4	0.828			
		I4.0 D5	0.846			
	I4.0 INT	I4.0 INT1	0.887	0.711	0.831	0.623
		I4.0 INT2	0.622			
		I4.0 INT3	0.731			
**2**^**nd**^ **Order Constructs**	**Variables**	**Path Coefficients (t-Value)**		**Cronbach’s Alpha**	**CR**	**AVE**
**CSR**	ECCSR	0.867 (35.235)		0.832	0.899	0.748
	ENCSR	0.872 (63.865)				
	SCSR	0.856 (45.090)				
**GIC**	HC	0.881 (61.931)		0.842	0.905	0.760
	RC	0.867 (44.506)				
	SC	0.867 (55.750)				
**SP**	ECP	0.869 (46.330)		0.820	0.893	0.735
	ENP	0.834 (40.778)				
	SCP	0.868 (42.728)				
**I4.0ES**	I4.0 D	0.917 (4.836)		792	859	753
	I4.0 INT	0.816 (15.220)				

The discriminant validity is analyzed using three criteria: HTMT ratio, Fornell Larker, and items cross-loadings. The results in Table 4 show that all the values are less than 0.90, thus meeting the HTMT ratio criteria.

**Table 4:** HTMT ratio.

Constructs	I4.0 D	I4.0 INT	ECCSR	ECP	ENCSR	ENP	GAI	HC	RC	SC	SCP	SCSR
I4.0 D												
I4.0 INT	**0.651**											
ECCSR	0.147	**0.092**										
ECP	0.289	0.200	**0.634**									
ENCSR	0.327	0.247	0.783	**0.772**								
ENP	0.328	0.275	0.590	0.683	**0.729**							
GAI	0.293	0.129	0.658	0.801	0.785	**0.751**						
HC	0.257	0.197	0.439	0.619	0.666	0.662	**0.746**					
RC	0.301	0.171	0.374	0.572	0.694	0.609	0.720	**0.802**				
SC	0.237	0.159	0.488	0.637	0.694	0.636	0.760	0.834	**0.778**			
SCP	0.302	0.182	0.570	0.798	0.733	0.692	0.766	0.588	0.566	**0.567**		
SCSR	0.191	0.150	0.714	0.644	0.723	0.578	0.652	0.536	0.556	0.615	**0.587**	

Similarly, Table 5 represents the values of the Fornell-Larker Criterion. All the values in the diagonal (square root of AVE) are higher than their vertical and horizontal values, thus fulfilling the criteria. Finally, item cross-loadings were checked, and the results confirmed that no item cross-loaded under other constructs.

**Table 5:** Fornell Larker Criterion.

Constructs	I4.0 D	I4.0 INT	ECCSR	ECP	ENCSR	ENP	GAI	HC	RC	SC	SCP	SCSR
I4.0 D	**0.794**											
I4.0 INT	0.518	**0.789**										
ECCSR	0.124	0.066	**0.843**									
ECP	0.249	0.167	0.545	**0.856**								
ENCSR	0.271	0.204	0.639	0.602	**0.748**							
ENP	0.300	0.235	0.515	0.565	0.572	**0.847**						
GAI	0.251	0.068	0.572	0.662	0.620	0.625	**0.764**					
HC	0.228	0.159	0.373	0.499	0.512	0.546	0.609	**0.791**				
RC	0.260	0.150	0.328	0.471	0.541	0.509	0.597	0.653	**0.809**			
SC	0.205	0.134	0.401	0.500	0.517	0.513	0.601	0.647	0.618	**0.764**		
SCP	0.264	0.158	0.498	0.664	0.580	0.577	0.644	0.484	0.472	0.449	**0.875**	
SCSR	0.179	0.126	0.638	0.554	0.590	0.501	0.568	0.458	0.483	0.506	0.510	**0.859**

Furthermore, the issue of lateral collinearity was evaluated by checking the VIFs of all LOCs. All VIFs are less than 5.0, indicating the model is collinearity-free [112]. We have checked the R^2^ and adjusted R^2^ and Q^2^ to assess the structural model’s predictive relevance [125]. R^2^ values of GIC, GAI, and SP are 0.375, 0.538, and 0.711, as represented in Table 6. The blindfolding technique was employed to assess Q^2^, and the values are 0.280 for GIC, 0.563 for GAI, and 0.507 for SP, indicating that the model has adequate predictive power [112].

**Table 6:** Total effect size and predictive relevance of the model.

Variables	R^2^-Explanatory Power	Adjusted R^2^	Q^2^-Predictive Relevance
GIC	0.375	0.373	0.280
GAI	0.583	0.581	0.563
SP	0.711	0.705	0.507

### 4.3 Assessment of the hypothesized model

**Fig 2:**
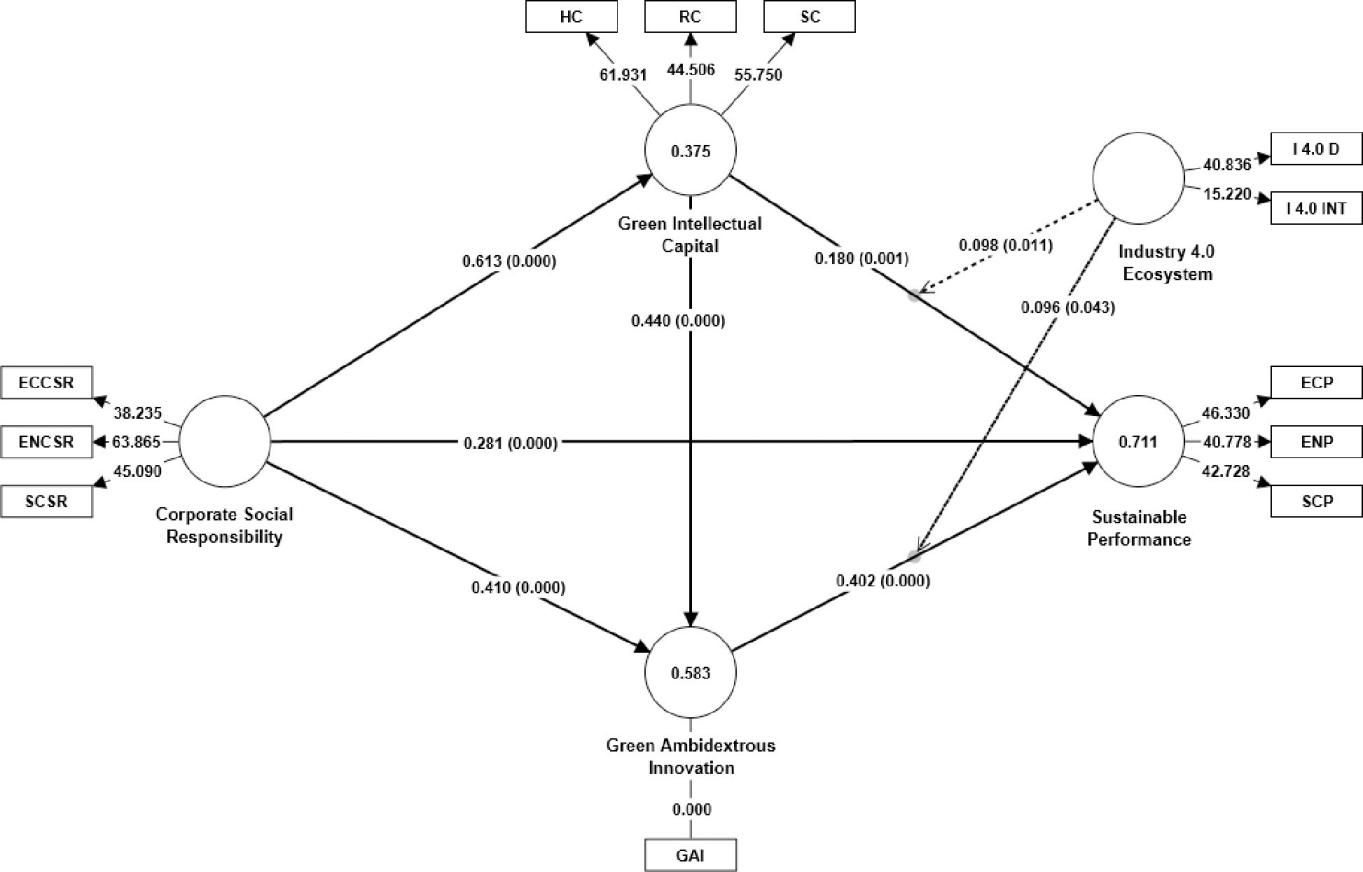
Hypothesized results.

The hypotheses were examined using the bootstrapping procedure with 5000 subsamples. The results are presented in Fig 2 and Table 7. The findings indicate that CSR is significantly associated with SP (β 0.281, t 6.010, p 0.000), confirming H1. Similarly, CSR exhibits a significant relationship with GIC (β 0.613, t 11.807, p 0.000), supporting H2a. The path coefficient between GIC and SP is also significant (β 0.180, t 3.329, p 0.001), confirming H2b. Additionally, CSR and GAI are found to be significant (β 0.410, t 4.029, p 0.000), supporting H3a.

**Table 7:** Path coefficients.

Hypotheses	Beta	Standard Deviation	t-Statistics	P-Values
**H1:** CSR -> SP	0.281	0.047	6.010	0.000
**H2a:** CSR -> GIC	0.613	0.052	11.807	0.000
**H2b:** GIC -> SP	0.180	0.054	3.329	0.001
**H3a:** CSR -> GAI	0.410	0.102	4.029	0.000
**H3b:** GAI -> SP	0.402	0.059	6.849	0.000
**H4:** GIC -> GAI	0.440	0.108	4.077	0.000

Furthermore, GAI significantly correlates with SP (β 0.402, t 6.849, p 0.000), supporting H3b. Additionally, the relationship between GIC and GAI is significant (β 0.440, t 4.077, p 0.000), validating H4.

The mediating and moderating analysis results are presented in Table 8. The first mediating path examines the indirect effect of CSR on SP through GIC. The results show a significant positive relationship (β 0.111, t 2.832, p 0.005), indicating that GIC mediates the relationship between CSR and SP, confirming H2c. The second mediating path assesses CSR’s indirect effect on SP through GAI. The results report a significant positive association (β 0.165, t 3.040, p 0.002), suggesting a significant mediation effect of GAI on the relationship between CSR and SP validating H3c. Moreover, the results also show that I4.0ES moderates the relationship between GIC and SP (β 0.098, t 2.552, p 0.011) and GAI and SP (β 0.096, t 2.026, p 0.043), confirming H5a and H5b.

**Table 8:** Indirect path.

Hypotheses	Beta	Standard Deviation	t-Statistics	P-Values
**H2c:** CSR -> GIC -> SP	0.111	0.039	2.832	0.005
**H3c:** CSR -> GAI -> SP	0.165	0.054	3.040	0.002
**H5a:** I4.0ES x GIC -> SP	0.098	0.038	2.552	0.011
**H5b:** I4.0ES x GAI -> SP	0.096	0.047	2.026	0.043

## 5. Discussion and conclusion

The importance of green knowledge and innovation has significantly amplified as SMEs strive to achieve SDGs. Green knowledge encompasses the specialized knowledge, expertise, and intellectual capital that enable businesses to navigate sustainability challenges and capitalize on emerging opportunities. This study draws upon NRBV and Stakeholders Theory to investigate a model in which CSR, GIC, and GAI trigger green synergy to uplift SP. Specifically, this study tested the mediating role of GIC and GAI in the relationship between CSR and SP. We collected the data from 317 SMEs in China and employed PLS-SEM using Smart-PLS V.4. This study’s findings align with the anticipated outcomes and provide valuable insights into the intricate connections between the relationships between CSR and SP, particularly with the mediating role of GIC and GAI. The results show that CSR is directly linked to SP, showing an indirect relationship between GIC and GAI. The findings show that a firm’s performance measurement should be tailored to its primary green drivers [49, 126]. It is consistent with previous studies suggesting that stakeholders’ increasing emphasis on CSR drives companies to incorporate social and environmental perspectives into their performance measurement systems [127, 128].

Furthermore, CSR significantly influences GIC and GAI. The significant relationships indicate that organizations prioritizing CSR activities are more likely to enhance their intellectual capital and foster ambidextrous innovation in sustainability. Chen [29] asserts that the presence of GIC enhances a firm’s chances of gaining competitive advantages. This finding aligns with prior research [69, 87], emphasizing the positive relationship between CSR and these constructs. The findings also show a positive relationship between GIC and, GAI and SP. These findings support the view that GIC and GAI are key drivers in improving SP. The results imply that organizations that effectively develop and utilize their intellectual capital while promoting ambidextrous innovation are more likely to achieve better SP outcomes. Several similarities are observed when comparing the findings of this study with those of previous literature. For instance, Chang and Chen [69] reported a positive relationship between CSR and GIC, which is consistent with our results. Additionally, Yusoff [15] found that GIC positively influenced SP, aligning with our findings. These consistent results across studies suggest the robustness and generalizability of the relationships between these relationships.

Finally, the study showed a positive moderating role of the I4.0ES between GIC, SP, and GAI and SP. Drawing upon stakeholders’ theory, this study confirms that external pressures and norms shape firms’ behavior and decision-making processes. SMEs are implementing I4.0ES technologies and trying to create an ecosystem due to stakeholders’ pressure. This results in a positive association between these factors and uplifting SP. Previous research failed to discuss the context of I4.0ES. Thus, this study fills the gap by exploring the context of I4.0ES, among other key factors such as CSR, GIC, GAI, and SP.

## 6. Theoretical and practical significance

### 6.1 Theoretical significance

This study’s findings have significant theoretical implications, particularly in the context of NRBV and Stakeholders theory. This research offers valuable insights into the relationships between CSR, GIC, and GAI within the framework of the I4.0ES in improving sustainability. The study contributes to the NRBV by highlighting the role of CSR as a strategic resource that can positively impact GIC, GAI, and SP. The findings confirm that SMEs focusing on CSR practices tend to improve their intellectual capital, improving ambidextrous innovation and SP. This aligns with the NRBV’s central premise of the strategic value of resources and their influence on SP. Moreover, the study provides insights in line with the SP, demonstrating that implementing CSR practices contributes to developing trust and social exchange between organizations and stakeholders. By adopting sustainable practices, firms provide intellectual capital, enhancing their ambidextrous innovation. The results suggest that CSR practices foster positive social exchanges, leading to higher innovation performance. Furthermore, the study extends the claims of SP by supporting the moderating role of I4.0ES between GIC and SP and GAI and SP. These results expand the perspectives of Stakeholder theory and bridge the relevant literature gap. This study also suggests and validates a Green Synergy Model, demonstrating how CSR, GIC, and GAI interact to uplift SP. This conceptual framework offers a theoretical basis for understanding the interrelation of green initiatives within organizations and their cumulative influence on sustainable outcomes.

### 6.2 Practical significance

This study provides managerial implications. First, managers underscore the significance of integrating CSR practices into the fundamental values of organizations. This arrangement directly influences SP and affects GIC and GAI, thus fostering a holistic approach to sustainability. Second, managers should regard GIC and GAI as valuable assets and focus on enhancing firms’ green knowledge of sustainability, encouraging collaboration, and generating a supportive environment for green innovation. Through this, they can build sustainable products and processes that contribute to SDGs and strengthen the organization’s competitive advantage in the long run. Third, as highlighted by the study, performance measurement systems should be tailored to reflect the organization’s primary green drivers. Organizations can effectively track and assess their progress towards sustainability goals by aligning performance metrics with CSR activities and green initiatives, optimizing resource allocation and decision-making processes. Fourth, policymakers must recognize the role of the I4.0ES in enhancing the association between GIC, GAI and sustainability, which produces a supportive ecosystem for sustainable innovation. Hence, government and regulatory bodies should provide supportive policies, incentives, and regulations that promote the responsible use of technology among SMEs to achieve sustainable goals.

Moreover, Chinese technology firms have efficiently leveraged their CSR initiatives to increase their intellectual capital and foster green innovation. By integrating advanced green technologies and sustainable practices into its operations, Huawei has substantially improved SP [129], setting a benchmark for other SMEs. This real-world example highlights the position of adopting a holistic approach that combines CSR, GIC, and GAI. By prioritizing these elements, SMEs can meet regulatory requirements and stakeholder expectations and gain competitive advantages in the market. This study suggests that SMEs should strategically invest in green knowledge and innovation, as demonstrated by industry leaders, to drive sustainable growth and performance.

## 7. Limitations and future research avenues

This study has certain limitations that offer opportunities for future research. First, it incorporates the cross-sectional approach, leaving uncertainty regarding whether CSR, GIC, GAI, and SP outcomes in small and medium enterprises provide identical outcomes in a longer time. Hence, future researchers can use the same research framework to observe whether outcomes change or remain similar over more extended periods. This study collected data from small and medium enterprises in China, and future scholars can collect data from the corporate sector and large-scale organizations to see the changes in results. Future researchers can also use green capability and green transformational leadership as a mediating construct between CSR and SP to observe whether it is significant. Finally, the current study was conducted in China, which has its own culture; future researchers can conduct a similar study in other countries to see the changes. Moreover, circular economy principles can be used to determine social, environmental, and economic performance.
